# The role of CXCR3 and its ligands expression in Brucellar spondylitis

**DOI:** 10.1186/s12865-020-00390-9

**Published:** 2020-11-18

**Authors:** Xin Hu, Xiaoqian Shang, Liang Wang, Jiahui Fan, Yue Wang, Jie Lv, Shaxika Nazierhan, Hao Wang, Jing Wang, Xiumin Ma

**Affiliations:** 1 State Key Laboratory of Pathogenesis, Prevention and Treatment of High Incidence Diseases in Central Asian, Clinical Laboratory Center, Tumor Hospital Affiliated to Xinjiang Medical University, Urumqi, Xinjiang 830011 P.R. China; 2 First Affiliated Hospital of Xinjiang Medical University, Urumqi, Xinjiang 830011 P.R. China; 3 Department of Spinal Surgery, People’s Hospital of Xinjiang Uygur Autonomous Region, Urumqi, Xinjiang 830011 P.R. China

**Keywords:** Brucellar spondylitis, IFN-γ, CXCR3, CXCL9, CXCL10

## Abstract

**Aim:**

Brucellar spondylitis (BS) is one of the most serious complications of brucellosis. CXCR3 is closely related to the severity of disease infection. This research aimed to study the degree of BS inflammatory damage through analyzing the expression levels of CXCR3 and its ligands (CXCL9 and CXCL10) in patients with BS.

**Methods:**

A total of 29 BS patients and 15 healthy controls were enrolled. Real-Time PCR was used to detect the mRNA expression levels of IFN-γ, CXCR3, CXCL9 and CXCL10 in peripheral blood mononuclear cells (PBMCs) of BS patients and healthy controls. Hematoxylin-Eosin staining was used to show the pathological changes in BS lesion tissues. Immunohistochemistry staining was used to show the protein expression levels of Brucella-Ab, IFN-γ, CXCR3, CXCL9 and CXCL10 in BS lesion tissues. At the same time, ELISA was used to detect the serum levels of IFN-γ, CXCL9 CXCL10 and autoantibodies against CXCR3 in patients with BS.

**Results:**

In lesion tissue of BS patients, it showed necrosis of cartilage, acute or chronic inflammatory infiltration. Brucella-Ab protein was abundantly expressed in close lesion tissue. And the protein expression levels of IFN-γ, CXCR3 and CXCL10 were highly expressed in close lesion tissue and serum of BS patients. At the same time, the mRNA expression levels of IFN-γ, CXCR3 and CXCL10 in PBMCs of BS patients were significantly higher than those in controls.

**Conclusion:**

In our research, the expression levels of IFN-γ, CXCR3 and its ligands were significantly higher than those in controls. It suggested that high expression levels of IFN-γ, CXCR3 and its ligands indicated a serious inflammatory damage in patients with BS.

## Introduction

*Brucella* is a facultative anaerobic intracellular bacterium that causes brucellosis in human. Brucellosis is a contagious zoonotic disease, also known as undulant fever. The *Brucella* infects human through direct or indirect contact with contaminated animal products (such as eating raw meat or unpasteurized dairy products) [1]. It is mainly distributed in Central America, South America, Africa and Asia [2]. It was listed as a class B infectious disease in China. Xinjiang is an agricultural region in northwest of China with abundant livestock products. The high morbidity percent of brucellosis is an average of 36.01 / 100,000 from 2014 to 2016 [3].

Brucellosis patients have non-specific symptoms, such as fever, fatigue, hyperhidrosis, myalgia, and joint pain [4]. Brucellosis can involve tissues and system but mainly affect joints and skeletal system [5]. According to reports, spondylitis is the most frequent complication of brucellosis and primarily affects the lumbar and thoracic vertebrae [6]. Back pain and sciatica are the most common complaints. In the existing environment, the diagnosis of brucellar spondylitis (BS) is still difficult. While blood culture of *Brucella* are the high accuracy for diagnosis brucellosis, the false negative rate of this method is still high. Thus, clinicians usually combine clinical manifestations (lower back pain, etc.), serological examination (IgM/IgG antibody levels) and imaging examination to diagnose BS.

Chemokines as a superfamily of chemotactic cytokines play an important role in leukocyte chemotaxis. *Brucella* infects the host will interfere with the host’s immune system and activate the function of immune cells [7]. Naive CD4^+^ T cells differentiate into T-helper type 1 (Th1) cells and T-helper type 2 (Th2). Th1 cells play a critical role against *Brucella* by secreting interferon-gamma (IFN-γ), interleukin-6 (IL-6) and tumor necrosis factor (TNF). C-X-C motif chemokine receptor 3 (CXCR3) is a G protein-coupled receptor [8]. IFN-γ can stimulate the production of ligands that induce CXCR3 [9]. The activation and migration of lymphocytes / mononuclear macrophages were regulated by receptor-ligand interactions. This effect plays an important role in immune regulation, inflammation and infection. CXCR3 ligands include C-X-C motif chemokine ligand 9 (CXCL9) and C-X-C motif chemokine ligand 10 (CXCL10).

In previous reports, BS caused serious damage to the body. High expression of IFN-γ has important clinical significance in brucellosis. Xu [10] reported that the increased level of IFN-γ and TNF-α in the serum of patients with acute brucellosis. Freeman [11] found that CXCR3 was significantly increased in severe chronic non-obstructive diseases. Pallett [12] reported that the CXCR3 gene was amplified in severe hepatitis B cells. Most patients have mild symptoms after *Brucella* infection, however, someone could recover after infection, while in others the spread of the infection causes severe brucellosis. With the development of infection, chemokines recruit a large number of inflammatory cells to the infection site. The infiltration of these inflammatory cells further aggravated the pathological damage. We analyzed the expression of CXCR3 and its ligands to explore the degree of inflammation damage in BS patients.

## Materials and methods

### Patients

This study is a descriptive observational. Patients diagnosed with BS were recruited in spine surgery at two hospitals in Xinjiang, Urumqi from 2015 to 2019. The study was approved by the ethics committee of Xinjiang Medical University. The patients were categorized as acute phase (with symptoms less than 3 months), subacute phase (3–6 months) and chronic phase (more than 6 months). The clinical symptoms and laboratory results were collected. The paravertebral cartilage tissue (include close and distant tissue) and peripheral blood of 29 BS patients were collected. 15 healthy controls were enrolled. The consent of patients was received at the same time. The data used to support the findings of this study are available from the corresponding author upon request.

The inclusion criteria of BS patients: 1. Patients have a history of contact with *Brucella* infected livestock or livestock products, consumption of raw meat and unpasteurized dairy products. 2. The patients have a persistent pain in the lower back, sacroiliac, fever, sweating, fatigue, or joint pain for days or even weeks. 3. *Brucella* was detected after one-week blood culture. 4. Tube agglutination test is positive. 5. Magnetic Resonance Imaging (MRI) reports that the adjacent surface of vertebra is destroyed, and there are hyperplasia and sclerosis. 6. Exclude patients with other immune, neoplastic diseases and HIV infection.

### Specimens

BS tissue specimen (*n* = 29) and corresponding peripheral blood samples (*n =* 29) was obtained. All samples were collected at the close and distant normal tissues, by which tissues in vitro were washed in PBS (Boster, CN), and fixed in 4% paraformaldehyde (Beijing Chemical Works, CN) for 24–48 h. Then the tissues were decalcified in 10% EDTA decalcification solution for 21–30 days. The samples were then embedded in paraffin and sectioned transversely at 4 μm continuously to make parallel sections. Peripheral blood (3 ml) was drawn from each subject and treated with Ficoll-Hypaque Solution (Tianjin TBD, CN) to get peripheral blood mononuclear cells (PBMCs).

### Hematoxylin-eosin (H&E) staining

H&E staining was performed according to routine procedure, including hematoxylin (Solarbio, CN) staining for 3 min, eosin (Solarbio, CN) staining for 2 min, 1% hydrochloric acid differentiation, ethanol gradient dehydration, neutral gum seal, and microscopic observation of histopathological changes.

### Immunohistochemistry (IHC) staining

In short, sections, 4 μm thick, were deparaffinised and hydrated. After antigen retrieval, they were treated with primary antibody (Dilution ratio: *Brucella*-Ab, 1:200; IFN-γ, 1:100; CXCR3, 1:200; CXCL9, 1:200; CXCL10, 1:200) (Concentration: *Brucella*-Ab, 5 μg/ml; IFN-γ, 10 μg/ml; CXCR3, 5 μg/ml; CXCL9, 5 μg/ml; CXCL10, 5 μg/ml) (Beijin Bioss, CN) at appropriate dilution at 4 °C overnight. Sections were incubated with secondary antibody (Zhongshan Jinqiao, CN) for 1.5 h. Visualisation was performed with 3, 3′-diaminobenzidine (DAB) as substrate, applied for 1.5 min. Sections were counterstained with Hematoxylin. Antigen expression was analyzed under × 20 medium power lens. Positive signals were required to meet two criteria: irregular patchy dark brown granules; more than five clusters. IHC positive expression area was processed by Image J. The average optical density (AOD) obtained was the final processing results.

### Real time PCR (RT-PCR)

Total RNA was extracted from the PBMCs by Trizol reagent (Invitrogen, USA). RNA quantity (> 80 ng/μl) and purity (OD260/OD280 = 1.8–2.0) were determined by nucleic acid quantifier. RNA was treated with DNase I (Takara, Japan) before reverse transcription to eliminate contaminating genomic DNA. cDNA was synthesized by reverse transcription according to the PrimeScript™ RT reagent Kit (Takara, Japan). The total amount of RNA was 3.75 μl. For RT-PCR, we mixed a 2 μl cDNA, 1 μl forward primers and 1 μl reverse primers (the final concentration of the primers was 10 μM.), 12.5 μl TB Green Premix Ex Taq (Takara, Japan) and 8.5 μl DEPC water. Thermal cycle parameters were: 95 °C for 30 s for 1 cycle, then 95 °C for 5 s, and 60 °C for 30 s for 39 cycles. Primer sequences are shown in Table 1. GAPDH was used as the internal control. The relative expression was calculated by the comparative cycle threshold method (2^-△△t^).

**Table 1:** Primer sequence

Gene name	Sequence	Genebank
IFN-γ		
Forward	CTAATTATTCGGTAACTGACTTGA	NM_000619.3
Reverse	ACAGTTCAGCCATCACTTGGA	
CXCR3		
Forward	ATGCGAGAGAAGCAGCCTTT	NM_001142797
Reverse	TCCTATAACTGTCCCCGCCA	
CXCL9		
Forward	GAAGCAGCCAAGTCGGTTAGTG	NM_002416.2
Reverse	AATCATCAGCAGTGTGAGCAGTG	
CXCL10		
Forward	TGGCATTCAAGGAGTACCTC	NM_001565.3
Reverse	TTGTAGCAATGATCTCAACACG	
GAPDH		
Forward	CATCCACTGGTGCTGCCAAGGCTGT	NM_001289745.2
Reverse	ACAACCTGGTCCTCAGTGTAGCCCA	

Abbreviations: *IFN-γ* interferon-gamma, *CXCR3* CXC chemokine receptor 3, *CXCL9* C-X-C motif chemokine ligand 9, *CXCL10* C-X-C motif chemokine ligand 10

### Enzyme-linked immuno sorbent assay (ELISA)

Serum levels of IFN-γ, CXCL9, CXCL10 (eBioscience, Austria) and autoantibodies against CXCR3 (CellTrend GmbH Luckenwalde, Germany), were detected in accordance with ELISA kit instructions. Standard wells, blank control wells and sample wells were set on 96-well plates. The diluted standard was added to the standard well, the sample dilution was added to the blank control well, and the test serum was added to the sample well. Then they were placed in 37 °C incubator for 60 min. After washing, color development and termination, the absorbance (A) was detected at a wavelength of 450 nm. For anti-CXCR-3-antibodies, the CXCR-3-receptor has been pre-coated onto a microtiter plate. During the first incubation the anti-CXCR-3-antibodies of the samples are immobilised on the plate. The autoantibodies are detected with a POD labeled antihuman IgG antibody. In the following enzymatic substrate reaction the intensity of the colour correlates with the concentration and/ or avidity of anti-CXCR-3-antibodies. The concentration was determined according to the standard curve.

### Statistical analysis

All data in this study were analyzed by SPSS 22.0 and GraphPad Prism 8.0. Quantitative data with normal distribution were expressed as mean ± standard deviation (SD) and t-test was used for comparison between groups. Quantitative data with a non-normal distribution are presented as medians (interquartile ranges), and the groups were compared using a Wilcoxon rank-sum test. The receiver operating characteristic (ROC) curve was established to evaluate the diagnostic value. The area under the curve (AUC) was calculated. The Youden index was used to determine the optimal cutoff value. A two-tailed *p* value of < 0.05 was considered to indicate significance.

## Results

### Clinical characteristics and laboratory results of BS patients

A total of 29 BS patients were enrolled in this study. Detailed demographic information was listed in Table 2.. Among them, 19 patients were male (65.5%) and 10 patients were female (34.5%), with an average age of 43.37 ± 18.27 years old (1 to 76 years old). There were 0 case in the acute phase, 1 case in the sub-acute phase, and 28 cases in the chronic phase. Eighteen patients (62.1%) were cattle and sheep herders, and 10 patients (34.5%) ate raw meat or unpasteurized dairy products.

**Table 2:** Demographics of patients with BS

	BS (%)	Control (%)
Number of patients	29	15
Male	19 (65.5)	9 (60)
Female	10 (34.5)	6 (40)
**Age**	43.37 ± 18.27	
1–18 years	4 (13.8)	0 (0)
19–45 years	11 (37.9)	9 (60)
45–60 years	10 (34.5)	4 (27)
60–76 years	4 (13.8)	2 (13)
**Staging**		
Acute	0 (0)	–
Sub-acute	1 (3.4)	–
Chronic	28 (96.5)	–
**Ethnicity**		
Han	9 (31.1)	5 (33)
Minority	20 (68.9)	10 (67)
**Medical history**		
Exposure and farming of cattle or sheep	18 (62.1)	3 (20)
Consumption history of raw meat or dairy	10 (34.5)	3 (20)
None	1 (3.4)	9 (60)
**History of spinal surgery**	1 (3.4)	0 (0)

The clinical symptoms and laboratory results of BS patients were showed in Table 3. Some patients showed hyperhidrosis (65.5%), fatigue (62%) and lower back pain (69%). Some patients showed joint pain (51.7%) and fever (55.1%). There were 20 patients (69%) showed anemia, 19 (65.5%) with increased erythrocyte sedimentation rate (ESR), 18 (62.1%) with increased C-reactive protein (CRP), 11 (37.9%) with reduced albumin, 10 (34.5%) with abnormal leukocytes and 2 (6.9%) with of thrombocytopenia. The agglutination test antibody was positive (≥1: 100) in 23 cases (79.3%) with highest antibody titer of 1: 1600. The above data showed that high ESR, CRP and agglutination test antibody positive (≥1: 100) were important laboratory indicators. BS showed a slice-like low-density shadow around the articular surface or the vertebral body, which was invasive bone destruction. The vertebral body signal of MRI lesions was non-specific, showing a slice-like abnormal signal. T1-weighted image (T1WI) showed low-signal, T2WI showed slightly high-signal, and the lipid-pressing sequence showed high-signal. The enhanced scan showed uneven enhancement.

**Table 3:** Clinical characteristics and laboratory results of patients with BS

Symptoms	Number of patients	%
Fever	16	55.1
Hyperhidrosis	19	65.5
Fatigue	18	62
Anorexia	8	27.5
Weight loss	12	41.4
Nausea	5	17.2
Upper back pain	6	20.7
Low back pain	20	69
Joint pain	15	51.7
Hepatomegaly	6	20.7
Splenomegaly	7	24.1
**Laboratory**		
Anemia (Hb: male < 140 g / l, Female < 120 g / l)	20	69
Lymphocytosis (lymphocytes > 50%)	6	20.7
Thrombocytopenia (platelets < 125 × 10^9^ /l)	2	6.9
Leukocytosis (white blood cells > 9.5 × 10^9^/ l)	5	17.2
Leukopenia (white blood cells < 3.5 × 10^9^/ l)	5	17.2
Increased CRP (> 8 mg/l)	18	62.1
Increased ESR (> 20 mm/h)	19	65.5
Albumin(< 40 g/l)	11	37.9
Positive amber red plate test	29	100
Positive standard tube agglutination test(≥1: 100)	23	79.3

Abbreviations: *Hb* hemoglobin, *CRP* C-reactive protein, *ESR* erythrocyte sedimentation rate;

### mRNA expression of IFN-γ, CXCR3 and its ligands in PBMCs of BS patients

**Fig. 1:**
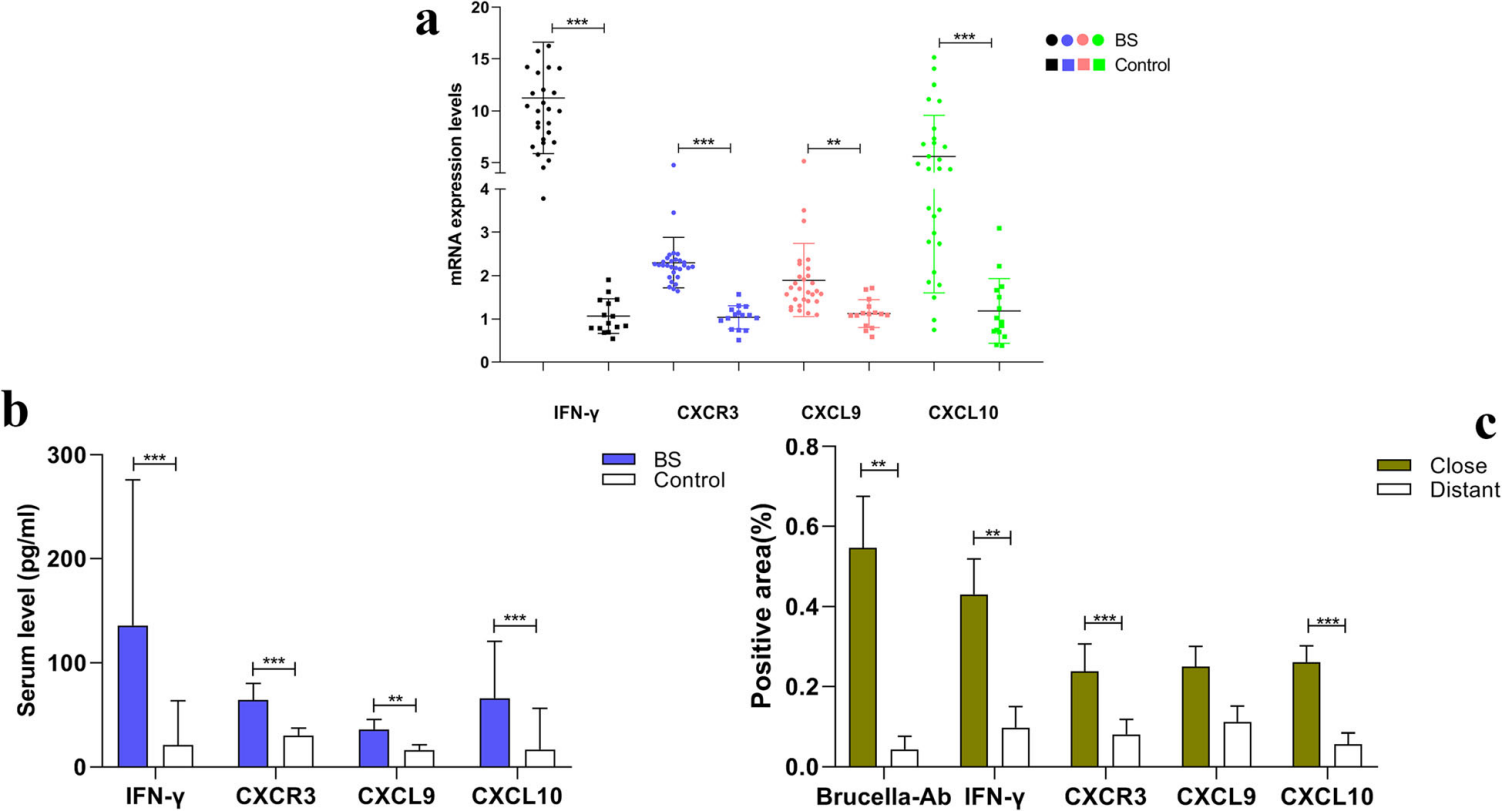
The expression of IFN-γ, CXCR3, CXCL9 and CXCL10 in PBMCs and tissues. **a** The mRNA levels of IFN-γ, CXCR3, CXCL9 and CXCL10 in PBMCs of 29 BS patients and 15 healthy controls. ^**^p < 0.01 or ^***^p < 0.001 were considered statistically significant. **b** The serum levels of IFN-γ, CXCL9, CXCL10 and autoantibodies against CXCR3 in 29 BS patients and 15 healthy controls. ^**^p < 0.01 or ^***^p < 0.001 were considered statistically significant. **c** Quantitative comparison of IFN-γ, CXCR3, CXCL9 and CXCL10 positive area. ^**^p < 0.01 or ^***^p < 0.001 were statistically significant

RT-PCR suggested that in PBMCs of BS patients had a higher mRNA expression: IFN-γ (11.24 ± 5.37, *p* < 0.001), CXCR3 (2.30 ± 0.58, *p <* 0.001), CXCL9 (2.37 ± 0.93, *p* < 0.01) and CXCL10 (6.02 ± 4.56, *p <* 0.001). Compared with IFN-γ (1.05 ± 0.40, *p <* 0.001), CXCR3 (1.04 ± 0.27, *p <* 0.001) and CXCL10 (1.18 ± 0.75, *p <* 0.001) in controls, the difference was statistically significant (Fig. 1a).

### ELISA levels of IFN-γ, CXCR3 and its ligands in serum of BS patients

We collected peripheral blood from 29 patients with BS and 15 healthy controls. The expression of CXCR3 related cytokines were analyzed from serum levels. IFN-γ are the main related factors of CXCR3. The levels of serum IFN-γ, CXCL9, CXCL10 and the levels of autoantibodies against CXCR3 in the BS patients were higher than control group, and the differences were statistically significant (*p <* 0.01) (Fig. 1b).

### Bone tissue and surrounding connective tissue of BS patients showed a highly protein expression of IFN-γ, CXCR3 and its ligands

**Fig. 2:**
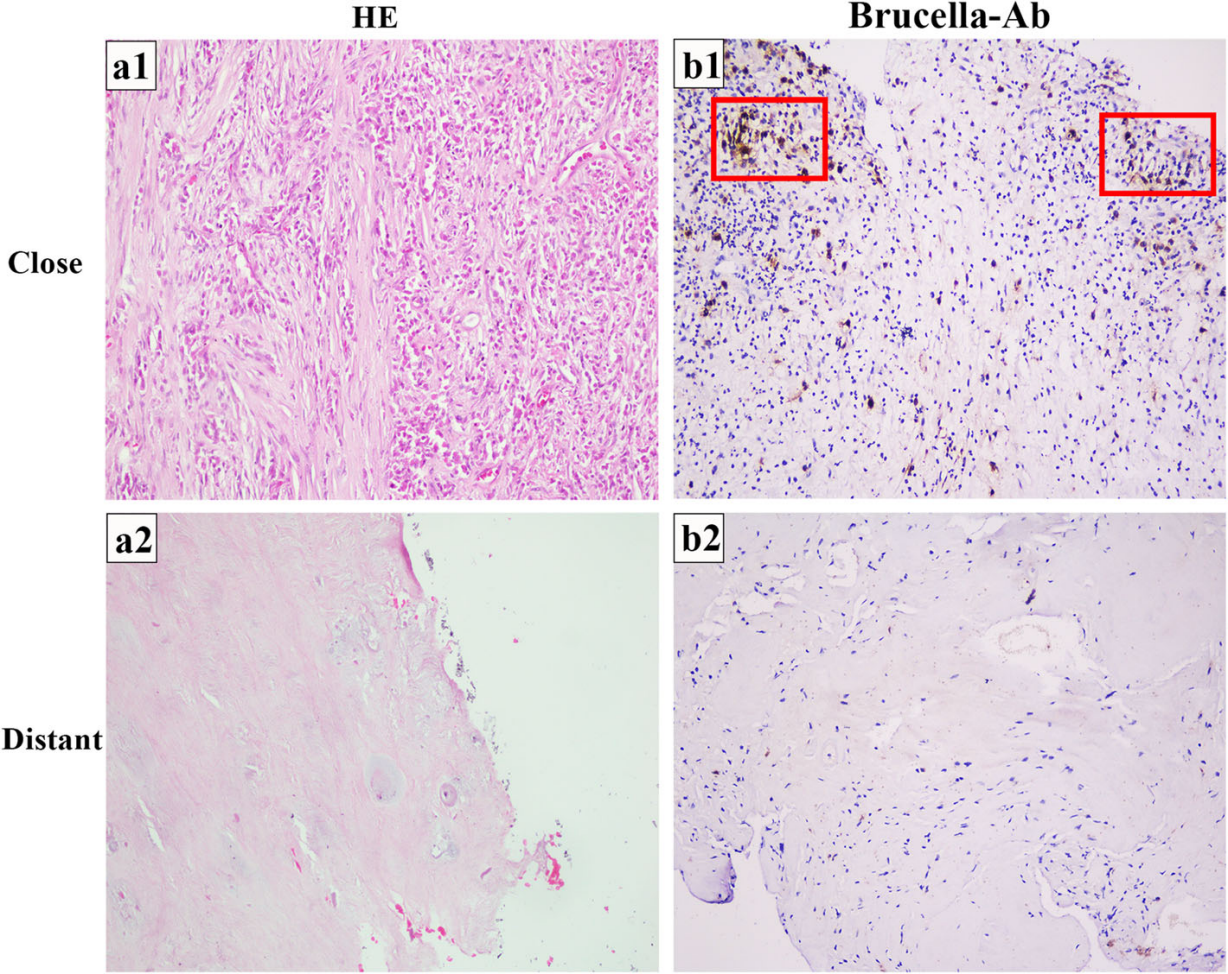
H&E staining in the close tissue and distant tissue of BS patients.**a**1 Close lesion tissue showed degenerated cartilage tissue. Fibrous tissue and small blood vessel hyperplasia, a mass of inflammatory cells infiltration. Visible new cartilage tissue and no obvious granuloma structure (20x). **a**2 Distant normal tissue of BS patients (20x). **b**1 Demonstration by IHC of *Brucella*-Ab in Close lesion tissue. Many macrophages show a strong positive cytoplasmic immunostaining (20x). **b**2 *Brucella*-Ab IHC staining of the distant normal tissue of BS patients showed negative results (20x)

At close lesion tissue of BS patients, there was significant degeneration of cartilage tissue. There was partial necrosis with a number of inflammatory cells infiltration in cartilage, fibrous tissue and small blood vessel hyperplasia. A minute amount of new cartilage tissue can be seen, no obvious granuloma (Fig. 2 a1). At the distant normal tissue, there were few or none inflammatory cells infiltration, and the cartilage tissue was normal (Fig. 2 a2).

In the lesion tissue, rabbit anti-human *Brucella*-Ab was used to detect the positive cells. It is suggested that the protein expression of inflammatory cells in close lesion tissue was strongly positive (Fig. 2 b1), while it was rarely or negatively expressed in the distant normal tissue (Fig. 2 b2). By comparing the Average Optical Density (AOD) of IHC results, the difference between the close (0.55 ± 0.13) and distant (0.04 ± 0.03) lesion tissue of *Brucella*-Ab expression was statistically significant (Fig. 1c).

**Fig. 3:**
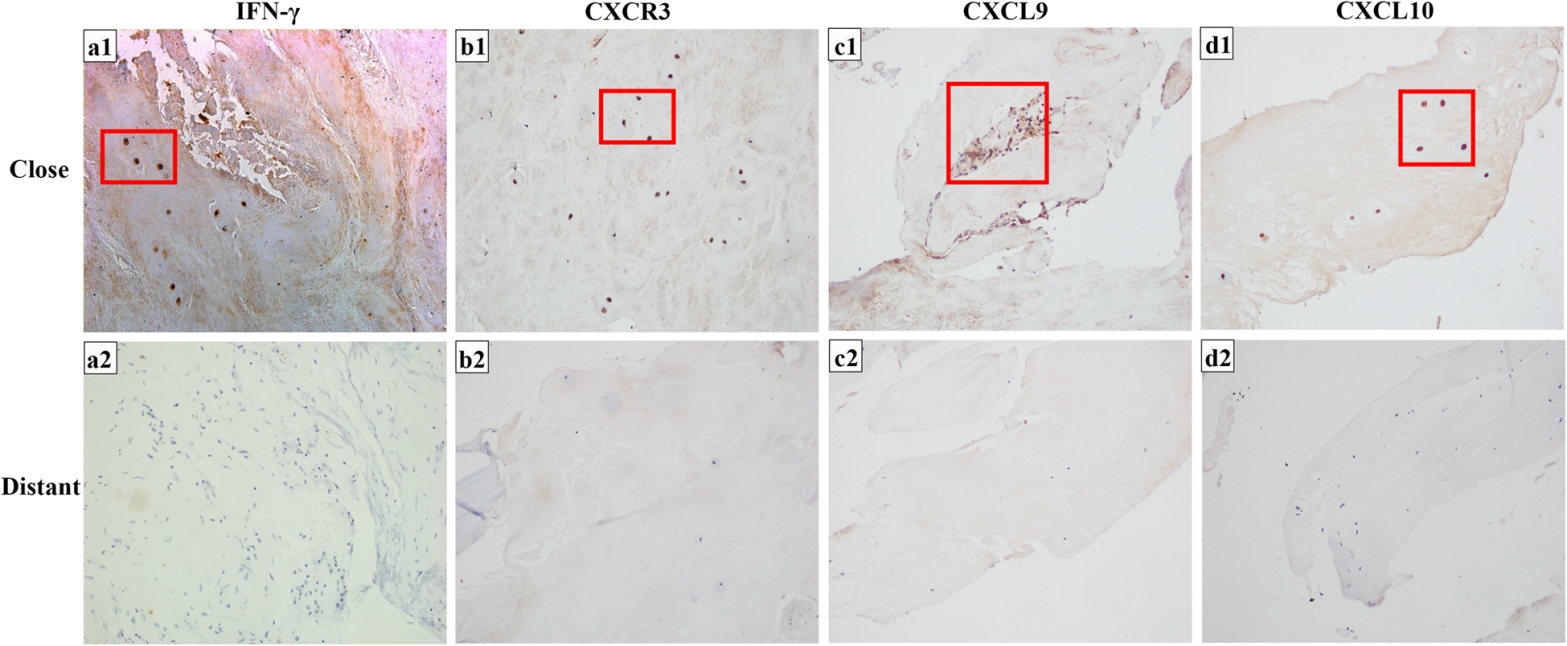
IHC staining in the close tissue and distant tissue of BS patients. **a**1 IFN-γ IHC staining of the close lesion tissue of BS patients showed that the positive cells were brown (20x). **a**2 IFN-γ IHC staining of the distant normal tissue of BS patients showed a small number of positive cells were pale yellow (20x). **b**1 CXCR3 IHC staining of the close lesion tissue of BS patients showed that the positive cells were brown (20x). **b**2 CXCR3 IHC staining of the distant normal tissue of BS patients showed a small number of positive cells were pale yellow (20x). **c**1 CXCL9 IHC staining of the close lesion tissue of BS patients showed that the positive cells were brown (20x). **c**2 CXCL9 IHC staining of the distant normal tissue of BS patients showed a small number of positive cells were pale yellow (20x). **d**1 CXCL10 IHC staining of the close lesion tissue of BS patients showed that the positive cells were brown (20x). **d**2 CXCL10 IHC staining of the distant normal tissue of BS patients showed a small number of positive cells were pale yellow (20x)

Immunohistochemistry (IHC) is the process of detecting antigens (e. g. proteins) in cells within a tissue section using specific antibodies. Interestingly, at the lesion tissue of BS patients, those markers not only expressed in immune cells but also in cytoplasm. The protein expression of IFN-γ (0.43 ± 0.09), CXCR3 (0.24 ± 0.07), CXCL9 (0.15 ± 0.05) and CXCL10 (0.26 ± 0.04) in the close lesion tissue were increased (Fig. 3 a1, 3b1, 3c1, 3d1). In distant normal tissue, IFN-γ (0.10 ± 0.05), CXCR3 (0.08 ± 0.04), CXCL9 (0.11 ± 0.03) and CXCL10 (0.06 ± 0.03) were slightly expressed (Fig. 3 a2, 3b2, 3c2, 3d2). The difference between IFN-γ, CXCR3 and CXCL10 was statistically significant (Fig. 1c).

## Discussion

Over recent years, the morbidity of BS has increased significantly, BS becomes a major health of the global population [13]. Xinjiang province is an animal husbandry region of China, the morbidity of brucellosis is high. However, BS still has an insufficient evidence for the diagnostic report. The increase in patients in this area will cause economic losses. In the past 5 years, the number of BS patients in our hospital has increased year by year.

In this study, 96.5% of BS patients were in the chronic phase, which was higher than the report of brucellosis [14]. It indicated that many patients in Xinjiang were not diagnosed timely. Epidemiologically, contacting with animals or consumption of uncooked, disinfected milk and dairy products are the major risk factors for infected persons [15]. The study showed that 34.5% of BS patients become infected by eating raw and uncooked dairy products and there are 62.1% of BS patients contacted with cattle or lambs, indicating that helping lambing is the main risk for infection.

After *Brucella* infects the human body, the endplate of the vertebral body, which have a rich blood supply, is the first infection vertebral bodies of *Brucella.* It invades the intervertebral disc and vertebral body, causing spondylodiscitis. 29 BS patients, the most common was lower back pain, followed by hyperhidrosis, fatigue and fever (Table 3). Bone and joint pain and significant weight loss could be observed in nearly half patients. The bone and joint damage of the patients prevented them from engaging in regular physical activity. Their life quality was lower than before. The most common findings on physical examination were hepatomegaly and splenomegaly, which accounted for one quarter of the patients. In this study, fever is not the main feature of BS patients, which is related to the chronic phase of disease (96.5%).

*Brucella* survives and reproduces in host phagocytic cells. After it infects the host, it survives and replicates by escaping the host immune system. The infection from the acute stage to the chronic stage is related to various factors, such as the type of bacteria, diagnosis time and immune system response, etc. [16]. After *Brucella* has been phagocytosed, the innate immune system attempts to clear it, the differentiation of Th1 cells activates the production of IFN-γ, and IFN-γ plays a key role in fighting intracellular bacteria infection [17].

This research found that the protein expression of IFN-γ is increased in the close tissue of BS patients, and the mRNA expression of IFN-γ in PBMCs is also increased. IFN-γ has multiple effects on the immune system [18], especially in the initial stages of many immune reactions. CXCR3 is predominantly expressed on Th1 lymphocytes, and its agonists CXCL9 and CXCL10 are IFN-γ-inducible chemokines that promote Th1 responses.

Seiler [19] found that CXCR3 affects granuloma formation after intracellular bacterium infection. In this study, we demonstrated that the CXCR3 and its ligands (CXCL9 and CXCL10) are highly expressed in *Brucella* infected tissues and PBMCs. Studies have shown that chemokine receptors and ligands interaction have played an important role in leukocyte migration to immune response sites [20].

CXCL10, as known as interferon-gamma-inducible protein 10 (IP-10), was identified as a chemokine secreted by several cells types in response to IFN-γ [21]. Figure 1c showed that IFN-γ and CXCL10 were expressed in the close lesion tissue of BS patients, and the nuclear stain were dark brown, which was different from the distant normal tissue. When *Brucella* infects host, CXCL10 gradient from *Brucella* infected tissues recruits CXCR3^+^ immune cells to regulate immune responses around the lesion tissue [22], aggravating the degree of inflammation in patients with BS.

CXCL9 was first obtained by Farber [23] using differential hybridization technology from mouse IFN-γ stimulated macrophage RAW 264.7. The CXCL9/CXCR3 axis regulates immune cell migration, differentiation, and activation [24]. Figure 1c showed that CXCL9 was slightly increased in the close lesion tissue compared with the distant. The expression levels of CXCL9 were further increased in the PBMCs and serum of BS patients (Fig. 1a, b).

Of course, our research has a few limitations. First, our sample size is small, the time period of collecting samples is relatively large. The morbidity of BS patients is only a small part of brucellosis. Secondly, those markers in other types of infectious disease were not observed in our study. In the next study, we will further explore those markers expression in other infectious spondylitis.

## Conclusion

The mechanism of bone destruction caused by BS is complicated. As a rare type of brucellosis, BS seriously affects human life quality during the infection period, it is getting more and more attention in Xinjiang. This study focus on analyzing the expression levels of CXCR3 and its IFN-γ-induced ligands (CXCL9 and CXCL10) in BS patient tissues and PBMCs. In summary, it was found that IFN-γ, CXCR3 and CXCL10 were highly expressed in the mRNA and protein of BS patient tissues and PBMCs. When Brucellosis invades the body, it causes an immune response and awakens the immune cells, those cells produce chemokines. They continuously infiltrate the inflammatory site, aggravate the inflammatory damage, and the repeated attacks are difficult to recover for BS patients. It is important to explore the interaction between CXCR3 and its ligands to better explain the degree of inflammatory damage of BS disease.

## Data Availability

The data used to support the findings of this study are available from the corresponding author (Xiumin Ma and Jing Wang) upon request.
